# Duchenne Muscular Dystrophy: From Diagnosis to Therapy

**DOI:** 10.3390/molecules201018168

**Published:** 2015-10-07

**Authors:** Maria Sofia Falzarano, Chiara Scotton, Chiara Passarelli, Alessandra Ferlini

**Affiliations:** 1 Unit of Medical Genetics, Department of Medical Sciences, University of Ferrara, Ferrara, 44121 Italy; E-Mail: sctchr@unife.it; 2 Bambino Gesù Hospital, Rome, 00146, Italy; E-Mail: psschr@unife.it

**Keywords:** dystrophin, molecular diagnosis, Antisense Oligonucleotides, antisense delivery, DMD therapy

## Abstract

Duchenne muscular dystrophy (DMD) is an X-linked inherited neuromuscular disorder due to mutations in the dystrophin gene. It is characterized by progressive muscle weakness and wasting due to the absence of dystrophin protein that causes degeneration of skeletal and cardiac muscle. The molecular diagnostic of DMD involves a deletions/duplications analysis performed by quantitative technique such as microarray-based comparative genomic hybridization (array-CGH), Multiple Ligation Probe Assay MLPA. Since traditional methods for detection of point mutations and other sequence variants require high cost and are time consuming, especially for a large gene like dystrophin, the use of next-generation sequencing (NGS) has become a useful tool available for clinical diagnosis. The dystrophin gene is large and finely regulated in terms of tissue expression, and RNA processing and editing includes a variety of fine tuned processes. At present, there are no effective treatments and the steroids are the only fully approved drugs used in DMD therapy able to slow disease progression. In the last years, an increasing variety of strategies have been studied as a possible therapeutic approach aimed to restore dystrophin production and to preserve muscle mass, ameliorating the DMD phenotype. RNA is the most studied target for the development of clinical strategies and Antisense Oligonucleotides (AONs) are the most used molecules for RNA modulation. The identification of delivery system to enhance the efficacy and to reduce the toxicity of AON is the main purpose in this area and nanomaterials are a very promising model as DNA/RNA molecules vectors. Dystrophinopathies therefore represent a pivotal field of investigation, which has opened novel avenues in molecular biology, medical genetics and novel therapeutic options.

## 1. Duchenne Muscular Dystrophy

Duchenne muscular dystrophy (DMD, MIM 310200) is the most prevalent neuromuscular disorders, affecting up to 1/3600 male births worldwide [1]. It is caused by mutations in the dystrophin gene on the X chromosome [2] and the clinical signs are not present at birth. The average age of diagnosis is usually at four years, when the first symptoms appear [3].

The disease progresses very quickly and usually the patients necessitate a wheelchair at the age of 10 years; the life expectancy has also been significantly extended by using corticosteroid treatment and higher standards of medical care as artificial respirators, although they die for cardiac and respiratory complications [4]. DMD patients develop a severe cardiomyopathy that generally manifests at about 10 years and is prevalent in most patients by 20 years of age [5].

Becker muscular dystrophy (BMD) is the milder form of dystrophinopathy, with an incidence of 1 in 18,518 male births. BMD typically presents later than DMD, between ages 5 and 15 years [1] and the severity or course varies among patients.

X-linked dilated cardiomyopathy (XLDCM), a different cardio-specific phenotype of dystrophinopathy, is characterized by congestive heart failure due to dilated cardiomyopathy and the disease occurs at age 10–20 years [6].

The dystrophin gene is the largest gene described in human and its full length messenger RNA is mainly expressed in skeletal and cardiac muscle, and also, with small amounts, in the brain. The DMD gene produces three full-length isoforms, through three independent promoters, in brain, muscle, and Purkinje cerebellar neuron, but many other isoforms are generated by alternative splicing events [7].

In healthy muscle, the dystrophin protein is localized on the intracellular surface of the sarcolemma along all the length of the myofibers [8], it assembles with the dystrophin-associated glycoprotein complex (DGC), presents on plasma membrane of myofibers (dystroglycan, sarcoglycan, and neuronal nitric oxide synthase), to form the dystrophin-associated glycoprotein complex (DGC). The essential function of dystrophin in the muscle is stabilizes the fibers during contractions by binding to F-actin with N-terminal domain and to β-dystroglycan with C-terminal domain, acting as abridging and anchoring protein [9,10].

DMD disease is associated with mutations as deletions (65%), duplications (6%–10%), small mutations (10%), or other smaller rearrangements [11] that interrupt the open reading frame of RNA. These mutations lead to a loss of dystrophin protein expression resulting in a severe muscle wasting, respiratory and cardiac failure and death before the age of 30 [12]. The reason is that the loss of dystrophin disrupts the DGC complex, causes membrane instability with increased susceptibility to injury, and fiber necrosis. Moreover, in DMD patients, the regenerative ability of myofibers is compromised probably due to chronic injury that induce satellite cells exhaustion [9] and replacement of muscle with fibroadipose tissue [10].

Studies in animal models as well as in humans have shown that the activation of pathological cascades (calcium influx, inflammatory immune cells infiltration, cytokines, and proteolytic enzymes activation) could aggravate DMD disease progression [13].

The most widely used animal model for DMD is the *mdx* mouse, which has a spontaneous point mutation in exon 23 that causes the absence of the dystrophin protein in the muscle. This model presents a DMD skeletal muscle phenotype with decline in force/power with age, elevated creatine kinase (CK) levels, muscle necrosis and respiratory problems due to degeneration of diaphragm [5]. While in humans DMD leads to an early loss of muscle functionality, *mdx* mice show very mild symptoms until old age, probably due to the presence of utrophin [14].

There is no cure available for DMD, and the current interventions are based on prevention and management of complications. However, the researchers are working hard to develop a possible therapy to reduce both primary and secondary pathologic effects [15].

## 2. Molecular Diagnosis

The most common molecular defect in the DMD gene is the deletion of one or more exons, occurring in 65% of DMD cases, while the duplication accounts for 6%–10% of cases. The remaining cases (approximately 25%) are due to small mutations (missense, nonsense, and splice site variations) small rearrangements (insertions/deletions, small inversion). However, a lower rate of cases (approximately less than 2%) is caused by complex rearrangements and deep intronic changes.

**Figure 1:**
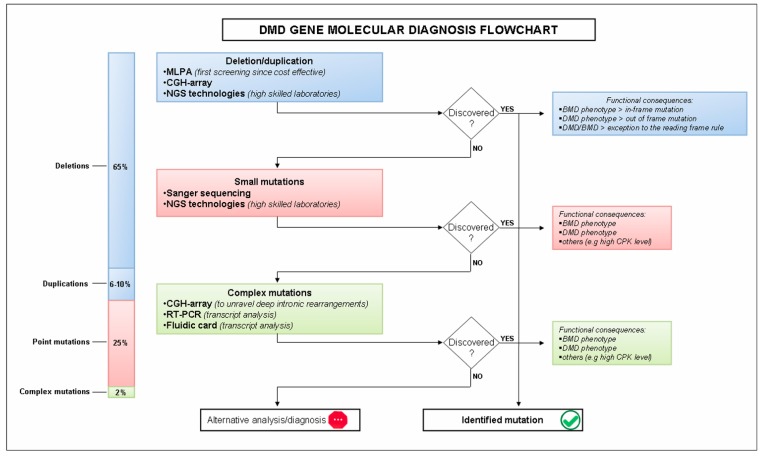
Flowchart of DMD diagnosis from suspicion to its confirmation. Procedures and tools for the identification of the mutation are shown. The phenotype depends on the type of mutation: DMD phenotype when the mutation alters the reading frame and leads to the complete absence of the dystrophin protein; and BMD when the mutation preserves the reading frame and allows translation of a partially functional dystrophin protein. Although, mutations that do not follow the reading frame rule are also identified.

The minimum level of diagnostic testing is designed for quantitative analysis of DMD genes to identify the majority changes of DMD gene, that are exons deletion or duplication, followed by qualitative approach represented by full gene sequencing (Figure 1).

Among the quantitative methods available, multiplex ligation-dependent probe amplification (MLPA) is currently the most widely used. This method, testing simultaneously all 79 exons of the DMD gene, identifies the copy number variation (CNV) in a multiplex polymerase chain reaction based reaction [16].

Another quantitative full-gene approach, which investigates the presence of CNVs in the entire genomic region of the DMD gene, is the oligonucleotide-based array comparative genomic hybridization (CGH). Although CGH was initially developed for cytogenetic analysis in order to detect chromosome unbalance, later other applications have been improved including the molecular genetics. The flexibility of CGH arrays is also due to the availability of custom arrays designed on the region of interest with the appropriate resolution. This gene-targeted CGH arrays have also been developed to study the DMD gene. This method allows to obtaining a full map of CNVs in the DMD gene, including intronic and 3′ and 5′ flanking regions, which are not investigated routinely. Therefore array-CGH is able to detect complex rearrangements and intronic alterations and more precisely define the mutation break-points [17,18]. The high density of probes in the array guarantees that mutations are detected by multiple probes, thus greatly reducing the possibility of false positives due to SNPs.

The qualitative analysis is represented by sequencing of the entire coding region of DMD gene in order to detect small mutations (small deletion or insertion, single base change, and splicing mutation).

A variety of technologies named next generation sequencing (NGS) emerged, each with a unique biochemical strategy. NGS introduced the concept of sequencing millions of copies of the DNA fragments simultaneously, increasing DNA sequencing output and reducing the time and cost necessary to fulfill the genetic diagnosis.

In general, these approaches use an *in vitro* clonal amplification or PCR step to amplify the DNA molecules present in the sample, followed by the alternative approaches of massively parallel sequencing based on pyrosequencing (454 by Roche Applied Science, Inc. Indianapolis, IN, USA), reversible dye-termination (Illumina, Inc. San Diego, CA, USA), sequencing by ligation (SOLiD by by Life Technologies, Inc. Carlsbad, CA, USA) and Ion semiconductor sequencing (Ion Torrent System by Life Technologies), as main examples. These platforms are currently being used in clinical laboratories for molecular diagnosis by a target capturing the regions of genomic interest; indeed different studies applying NGS have proven its ability in detecting small mutations in the DMD gene [16,19].

The implementation of NGS technology could lead to the development of a unique diagnostic method, indeed specific computational framework could drive the identification of CNV and single nucleotide variations at the same time [20,21].

This diagnostic workflow may not identify the 2% of complex rearrangements or deep intronic changes (Figure 1), therefore the RNA analysis of muscle tissue using RT-PCR and sequencing could become necessary to archive a genetic diagnosis in this subtype of rare mutations. An innovative diagnostic approach, able to simultaneously analyze DMD exon junctions using unique TaqMan Real-Time systems, have been recently reported [22]. This approach is new, accurate, rapid gene/exome-specific and could be applied to RNA extracted from a variety of patients’ tissues (muscle, skin, and cells) avoiding the invasive procedure. This method is able to detect CNVs and small mutations affecting the exon composition and to identify mRNA decay changes of the DMD transcript in patients with nonsense or out-of-frame mutations.

To summarize, the optimum procedures for DMD molecular diagnosis consist in quantitative analysis, to detect CNVs, followed by genomic sequencing or alternatively by NGS strategy. If this is still negative, transcript analysis should be performed to identify the genotype in the low percentage of undiagnosed patients.

## 3. Therapeutic Interventions

### 3.1. Corticosteroid

The gold standard therapy that can delay DMD progression is based on corticosteroids, which was first suggested in 1974 [23] and tested in several trials to define the optimal dose, age of initiation and frequency [24,25,26,27,28]. At present, the corticosteroids are the only pharmacologic agents with documented benefits, even if they are associated to several adverse effects (weight gain, Cushingoid appearance, nervous system disturbance, gastrointestinal symptoms, metabolic disorders, osteoporosis with increased risk of vertebral fractures), which can be reduced finding out an optimal standard regimen [29,30]. The two corticosteroids mainly used in DMD treatment are Prednisone/Prednisolone and Deflazacort, an oxazoline derivative of prednisolone, administered by two common regimens: daily and intermittent. The three regimens in most common use are 0.75 mg/day prednisone, 0.9 mg/kg/day deflazacort, and 0.75 mg/kg/day prednisone for 10 days on and 10 days off [31]. Both drugs have been equally effective in the short-term treatment trials (six months to two years), improving muscle strength and function and presenting adverse effects not considered clinically severe [24,25,26,27,28]. Moreover, in nonrandomized trials, significant beneficial effects on ambulation and cardiac function, delayed onset of both scoliosis and respiratory dysfunction, and a general amelioration of quality of life, have been observed after treatments for longer than two years with prednisone or deflazacort [32,33,34,35,36,37].

The mechanisms of action of corticosteroids in DMD, although not yet completely understood, have been widely studied in *mdx*. Prednisone and prednisolone show an anti-inflammatory effect [38], and, while having no influence on fibers regeneration, increase the force of skeletal muscle. In *mdx*, prednisolone prolonged life expectancy, but this observation is not directly comparable to human patients because of the different life-span of mouse model. Deflazacort acts on muscle regeneration and differentiation [39], reducing the skeletal muscle pathology [40]. On the other hand, adverse effects on cardiac muscle (increased fibrosis) observed in *mdx* mice after long-term treatments suggest that *mdx* could be an inappropriate positive control in long-term pharmacological studies [41].

Investigations on corticosteroids therapy have to take into account that a subset of patients do not tolerate chronic use of these drugs or show a lower responsiveness to treatment, while others seem to respond better [42]. Several studies are now focusing on identification of polymorphisms that could influence the efficacy of corticosteroids treatment. For example, polymorphisms at the amino acid position 363 (N363) of the intracellular glucocorticoid receptor (GRL), may have a role in corticosteroid sensitivity in some DMD patients [43,44].

By investigating the molecular mechanisms of glucocorticoids, Heier and collaborators found a novel oral drug, VBP15, designed for NF-κB inhibition, membrane insertion and glucocorticoid receptor specificity. It has been shown that VBP15 protects and promotes *in vitro* the skeletal muscle cells reparation and ameliorates in DMD model mice the pathology phenotypes independent of hormonal, growth, or immunosuppressive effects [45].

### 3.2. Novel Classical Pharmacological Approaches

Another pharmacological strategy is to target the signals downstream to the genetic defect such as for Givinostat (originally ITF2357), a histone deacetylase inhibitor with potential anti-inflammatory, anti-angiogenic, and antineoplastic activities. It has been demonstrated that histone deacetylase inhibitors slow disease progression in mdx mice, increase cross-sectional area of myofibers and reduce fibrotic tissue and fatty infiltration [46,47].

Givinostat is in phase II DMD clinical trial “to assess the safety and tolerability, PK, effects on histology and some clinical parameters” in ambulant DMD children [48].

The pathophysiology of DMD is also characterized by an altered synthesis of nitric oxide (NO) due to a reduction of NO synthase (nNOS) activity. Restoration of NO by NO donating drugs, administered together with nonsteroidal anti-inflammatory drugs (NSAID), induces persistent beneficial effect by reducing inflammation, enhancing activity of endogenous stem cells and preventing muscle wasting with recovery of skeletal muscle morphology and function [49].

A pilot study in adult dystrophic patients to assess the safety and tolerability of isosorbide dinitrate, a nitric oxide donor, co-administered with ibuprofen, a non steroid anti-inflammatory drug, showed high safety and tolerability profiles for a long-term treatment [50].

### 3.3. Cell Therapy

Cell based-therapies offers an opportunity to replace dystrophin for a potential cure, and stem cells are promising approach for the treatment of DMD, currently tested in Phase I/II clinical trial (EudraCT #2011-000176-33, [51]).

Previous studies performed in DMD patients using local intramuscular injection of myoblasts derived from healthy donors were not successful: the major limitations were the low survivability and migration, and the immune rejection of transplanted cells [52].

Stem cells are an alternative and promising approach for muscle regeneration for their ability to self-renew and differentiate into several cell types [53].

Different types of mesoderm-derived stem/progenitor cells were isolated and tested for their ability to differentiate into myogenic cells [52].

Mesoangioblasts (MABs), myogenic vessel-associated stem/progenitor cells, showed a good ability to cross the blood vessel wall and lead skeletal muscle regeneration with amelioration of muscular dystrophies phenotype in different pre-clinical animal models (*mdx* mouse and golden retriever dog for DMD, AJ mouse model for dysferlinopathy, α-sarcoglican-null mice for limb-girdle muscular dystrophy). Based on these pre-clinical analyses, human MAB from adult skeletal muscle are being tested in clinical trial based upon intra-arterial allogeneic transplantation [51].

### 3.4. Gene Therapy

A potential treatment for most DMD patients is the gene therapy for the delivery of a therapeutic gene to skeletal and cardiac muscle, in order to restore the dystrophin protein.

The dystrophin gene, however, is too large to fit into a recombinant adeno-associated virus (rAAV), the selected vector for DMD gene delivery due to its persistence in the muscle and absence of pathogenicity. For this reason, a different reduced-size dystrophin version (mini- or microdystrophin) based on observation that, in BMD patients, a truncated dystrophin leads a mild phenotype has been developed [52,54].

Previous studies reported that the intramuscular injections of mini-dystrophin delivered by AAV result in low levels of local dystrophin restoration and in an immunogenicity of the transgene product [55,56].

Different AAV serotypes were tested in order to ameliorate the limited carrying capacity of AAV and reduce the immune responses against the vector and the transgene product.

rAAV2/AAV8, rAAV6, rAAV9 showed promising for the mini- or micro-dystrophin deliver. In particular, rAAV6 and rAAV9 microdystrophin were able to induce dystrophin expression in skeletal muscle and heart of both young and old *mdx* mice, suggesting that the treatment is also effective in an old damaged muscle. Despite these good results, the major obstacle was the host immune response. To overcome this problem, rAAV2/8, a chimeric vectors, was developed and tested on *mdx* mice resulting effective to restore dystrophin expression in skeletal muscle and heart without immunological response [5].

### 3.5. Stop-Codon Read-Through: Mutation Suppression

About 15% of DMD patients have a premature stop codons that lead to a premature cessation of protein translation with resultant truncated and nonfunctional proteins. Aminoglycosides, a class of antibacterial drugs used for Gram-negative bacterial infections, bind to the decoding site of ribosomal RNA of both prokaryotes and eukaryotes, interfere with stop codons by introducing a nucleotide sequence at the aminoacyl transfer RNA acceptor site, allowing the expression of a full-length functional dystrophin protein [29].

The aminoglycosides present numerous limitations due to their side effects. The high dosage and the chronic treatment result in ototoxicity and renal toxicity. Other compounds were developed with different structure from aminoglycosides in order to identify other molecules with the same activity and high safety profiles [57]. PTC124 (Ataluren, now Tranlsarna™) is one of the identified agent, it is an oral nonaminoglycosides drug which has read-through activity with no antibiotic properties. The activity of Ataluren has been demonstrated in multiple disease models in which are involved many different organ systems such as dystrophinopathy. Preclinical studies in the *mdx* mouse demonstrated dystrophin expression in skeletal muscles, hearth, and diaphragm.

Two Phase 2a trials demonstrated that ataluren produced dystrophin in DMD patients with nonsense mutation and induced the expression of transmembrane conductance regulator (cftr) protein in nonsense mutation cystic fibrosis patients. A phase 2b study (randomized, double-blind, placebo-controlled) showed the efficacy and safety (well tolerated) of two doses of ataluren in patients with nonsense mutation dystrophinopathy [58].

“Ataluren was granted conditional marketing authorization in the European Union under the trade name Translarna™ for the treatment of nmDMD in ambulatory patients aged five years and older. Translarna is the first treatment approved for the underlying cause of DMD. The European Medicines Agency, or EMA, has designated ataluren as an orphan medicinal product and the U.S. Food and Drug Administration, or FDA, has granted orphan drug designation to ataluren for the treatment of both nmDMD and nmCF” [59].

### 3.6. Utrophin Modulation

Utrophin up-regulation was one of the first approaches to replace the dystrophin.

Utrophin is the homologue of dystrophin with a molecular weight of 395 kDa and with similar structural organization and protein binding properties. Utrophin is ubiquitously localized at the sarcolemma in *utero* and is progressively replaced by dystrophin when the muscle matures. In adult muscle, utrophin (A isoform) is localized to the neuromuscular and myotendinous junctions. In repairing muscle like in DMD patients and *mdx* mice, utrophin expression is naturally increased due to the absence of dystrophin in order to re-establish the continuity of the myotubes [6,60].

Despite some different functional characteristics between dystrophin and utrophin, it has been demonstrated that the utrophin expression increases with age in DMD, delaying the age of wheelchair [61] and utrophin can act as an effective substitute for dystrophin in *mdx* muscles [62].

Tinsley and collaborators developed an orally bioavailable small molecule, SMT C1100 (2-arylbenzoxazole (5-(ethyl sulfonyl)-2-(naphthalen-2-yl) benzo(d)oxazole)), after an exhaustive chemical screening and optimization. *In vitro* studies in human cells treated with SMT C1100 demonstrated that this molecule is an utrophin modulator able to increase utrophin mRNA and protein levels. These data were confirmed in different *in vivo* studies (*mdx* mice) that reported a reduction of dystrophic phenotype and an amelioration of muscle functions in both sedentary and the more severely affected forced exercise *mdx* model. The presence of utrophin protein was detected also in heart and diaphragm muscles [63].

Safety, tolerability, and pharmacokinetics studies of SMT C1100 in healthy volunteers showed that this drug is safe and well tolerated in healthy volunteers (Phase Ia study, 60). Phase Ib clinical trials to determine whether increasing doses of SMT C1100 are safe, well tolerated and achieve appropriate blood levels in patients with DMD has been completed but the results are not yet published [64].

### 3.7. Antisense Oligonucleotides and Exon Skipping

The idea for the development of splicing therapy for DMD to skip the exons with mutation on pre-mRNA transcripts derived from several observations on: (i) the modulation using antisense oligonucleotides of β-globin gene associated with β-thalassaemia; (ii) the presence of revertant fibers (dystrophin-positive) in DMD patients, due to intrinsic alternative splicing; and (iii) the shorter but functional dystrophin proteins found in the milder form of Becker muscular dystrophy (BMD) [65].

The antisense oligonucleotides (AONs) are 20–30 nucleotides in length, designed to target specific pre-mRNA sequences and to skip a specific DMD exon flanking the region of mutation, producing an in-frame but truncated transcript that translate a functional dystrophin protein [6].

**Figure 2:**
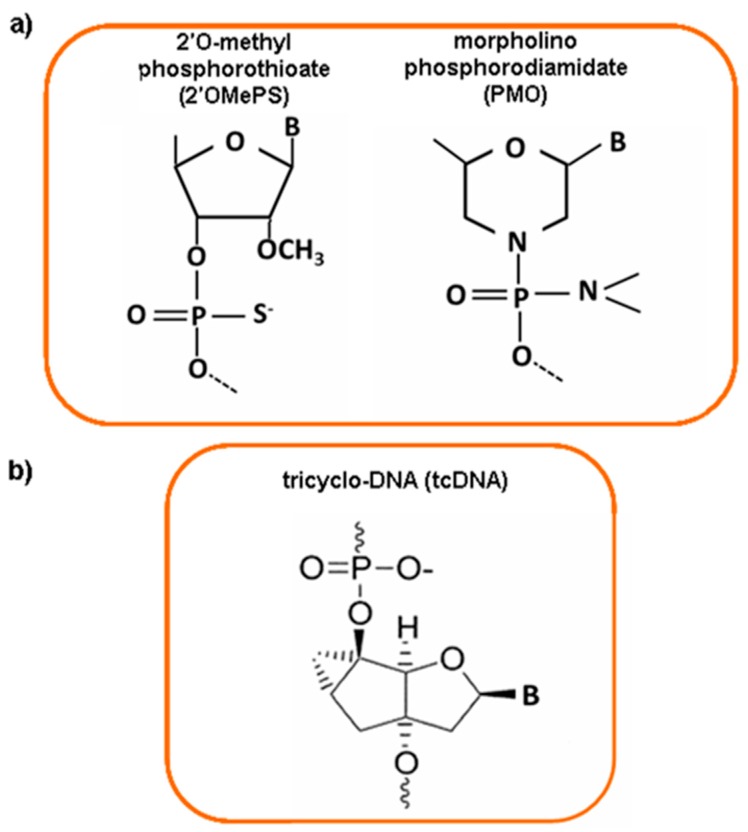
Chemical structure of different antisense oligonucleotide molecules. (**a**) 2′-O-methyl phosporothioate antisense (2′OMePS) and morpholino phosphorodiamidate oligomer (PMO); (**b**) tricyclo-DNA (tcDNA) compound.

Different AONs chemistry has been developed in order to confer them a nuclease resistance, without interfering with their ability to hybridize with the right target. Phosphorothioate oligonucleotides (PS) and morpholino phosphorodiamidate oligomers (PMO) are the most studied AONs. In PS oligonucleotides, nuclease resistance is conferred by replacing the non-bridging oxygen atom of the RNA phosphate group with a sulfur atom and by 2′-*O*-modification of the ribose residue (Figure 2a). The negative charge increases their solubility and allows them to complex with cationic lipids and proteins. PMO have a nonionic backbone at physiological pH, a ribose sugar replaced by a six-membered morpholine moiety, and phosphorodiester intersubunit bonds with phosphorodiamidate linkages (Figure 2a). PMO do not activate RNase H and their nonionic properties prevent potential nonspecific interactions with cellular components. It is possible to conjugate PMOs with different delivery systems, such as cell penetrating peptides (CPPs) [14].

The chemistries 2′*O*-methyl phosphorothioate (2′OMePS, named PRO051/Drisapersen initiated by Prosensa/GSK) and the PMO (named Eteplirsen initiated by AVI Biopharma, now Sarepta Therapeutics) are currently used in clinical trials [66]. Both AONs are designed to skip dystrophin exon 51 and they are able to restore the dystrophin protein after local injection [67,68] and systemic delivery [10,69,70].

Although the results were promising, recent clinical studies report that sufficient clinical benefit has not yet been achieved, probably due to a poor tissue uptake and low rescue of dystrophin expression [66]. For this reason, the progress of antisense therapy is dependent upon modifications in AONs chemistry in order to improve the delivery to target tissues and avoid the clearance by kidney and/or liver. Naked AONs do not enter the heart or cross the blood-brain barrier and the key challenge for the success of antisense approach is discover new molecules effective on cardiac muscle and central nervous system (CNS) for treating many multisystemic disorders [71]. Many different modifications are available as potential drugs for exon skipping [72]. A new class of AONs, named tricyclo-DNA (tcDNA), showed good uptake properties by many tissues, after systemic administration, in two mouse models of DMD. TcDNA-AON is an oligonucleotide analog of natural DNA with three additional carbon atoms between C5′ and C3′ (Figure 2b). This chemical modification increases RNA affinity, hydrophobicity, nuclease resistance for both phosphate and thiophosphate internucleotide linkages, and tcDNA-AONs do not activate RNase H. Systemic delivery of tcDNA-AONs induce high dystrophin rescue in skeletal muscles, heart and low levels in the brain, with amelioration of cardio-respiratory functions and DMD phenotype. Unlike the 2′OMe and PMO oligomers, tcDNA-AONs spontaneously form nanoparticles with size of 40–100 nm such as transfection reagents or nanoparticles used for the AONs delivery, improving cellular uptake compared to 2′OMe and PMO. These features make tcDNA-AON a very attractive tool for the future of the DMD therapy or for other diseases that need a systemic treatment with antisense molecules [71].

## 4. Nanoparticles as Delivery System for DMD Therapy

The success of nucleic acid therapies for DMD and other diseases requires the implementation of delivery platforms able to drive DNA/RNA molecules into target cell, overcoming challenges such as enzymatic degradation, low intracellular uptake and lysosomal entrapment [73]. At the present, although the massive efforts done for searching optimal delivery systems, many disadvantages are still present for their clinical use, for example an absent cell specificity and the difficulties in controlling drug release [74].

In the last years, interdisciplinary approaches combining nanotechnologies and biology are rapidly emerged as a precious tool for a better understanding of physio-pathologic mechanisms of diseases and for the development of biomedical applications. The nanomedicine, using nano-size technologies for prevention, diagnosis and treatment of diseases, is continuously searching for the optimal nanocarrier able to overcome limits of drug delivery. Many are the classes, with different size ranges and various physicochemical features, tested as non-viral delivery systems for drugs and macromolecules, such as plasmid DNA, oligonucleotides and proteins, through several administration routes (intramuscular, intravenous, oral, intraperitoneal, and mucosal) [75,76,77,78,79,80,81]. On the basis of their use, the most common nanodevices, differing in size and chemical properties, are: gold nanoparticles (5–50 nm), block copolymer micelles (50–200 nm), liposomes (80–200 nm), nanoparticles (20–1000 nm) and nanosized drug crystals (100–1000 nm) [82]. The ideal delivery system has to: (i) form stable complexes with nucleic acid/drug; (ii) be able to spread via systemic circulation; (iii) reach target tissues; and (iv) release the nucleic acid/drug in a controlled manner.

Over the past few years, many synthetic nanoformulations, such as liposomes, micelles and nanoparticles, have been tested in order to identify proper carriers for therapeutic molecules delivery. The usefulness of these new nanoparticles is mainly due to their ability to adapt to both nucleic acids and small molecules, but also to their safety profile [83].

The first nanoformulations approved by US Food and Drug Administration, about 25 years ago, were based on biodegradable poly(d,l-lactide) (PLA) and poly(ethylene glycol) (PEG) and poly(d,l-lactide-*co*-glycolide) (PLGA) polymers. Especially PLGA nanoparticles have been used for several applications, such as gene silencing by siRNA [84] or treatment of cerebral disorders [85].

Another nanoformulation widely used in the clinic or approaching the clinic are liposomes, which are considered an efficient delivery system, especially because of their high biocompatibility, biodegradability and propensity for size and surface manipulations [86]. For these reasons several types of liposomal drugs have been already approved and commercialized [87,88].

## 5. Conclusions

Although the molecular diagnosis of DMD is demanding, remarkable progress has been made over the last years resulting in exhaustive molecular testing able to discover multiple types of mutations (deletion/duplication, point mutation and deep intronic events). Accurate diagnosis using new high-throughput technologies such as CGH and NGS, will lead to an early and more effective intervention for patients.

The challenge of DMD treatment is the discovery of most safe and effective molecules. Currently, corticosteroid is the only available treatment for all DMD patients but its effect is not resolutive.

The knowledge of dystrophin patho-physio mechanisms and the synthesis of new molecules and delivery systems (nanomaterials) that overcome the stability, degradation and target specificity problems will allow the development of novel drugs for a promising DMD therapy.
